# Safety metric profiling in surgery for temporal glioblastoma: lobectomy as a supra-total resection regime preserves perioperative standard quality rates

**DOI:** 10.1007/s11060-020-03629-y

**Published:** 2020-09-29

**Authors:** Matthias Schneider, Inja Ilic, Anna-Laura Potthoff, Motaz Hamed, Niklas Schäfer, Markus Velten, Erdem Güresir, Ulrich Herrlinger, Valeri Borger, Hartmut Vatter, Patrick Schuss

**Affiliations:** 1 Department of Neurosurgery, University Hospital Bonn, Venusberg-Campus 1, 53127 Bonn, Germany; 2 Division of Clinical Neurooncology, Department of Neurology, University Hospital Bonn, Bonn, Germany; 3 Department of Anesthesiology, University Hospital Bonn, Bonn, Germany

**Keywords:** Anterior temporal lobectomy, Gross total resection, Postoperative complication profiling, Temporal glioblastoma surgery

## Abstract

**Introduction:**

Supra-total resection in terms of anterior temporal lobectomy (ATL) has gained growing attention with regard to superior long-term disease control for temporal-located glioblastoma. However, aggressive onco-surgical approaches—geared beyond conventional gross total resections (GTR)—may be associated with peri- and postoperative unfavorable events which significantly worsen initial favorable postoperative outcome. In the current study we analyzed our institutional database with regard to patient safety indicators (PSIs), hospital-acquired conditions (HACs) and specific cranial surgery-related complications (CSC) as high standard quality metric profiles in patients that had undergone surgery for temporal glioblastoma.

**Methods:**

Between 2012 and 2018, 61 patients with temporal glioblastoma underwent GTR or temporal lobectomy at the authors’ institution. Both groups of differing resection modalities were analyzed with regard to the incidence of PSIs, HACs and CSCs.

**Results:**

Overall, we found 6 PSI and 2 HAC events. Postoperative hemorrhage (3 out of 61 patients; 5%) and catheter-associated urinary tract infection (2 out 61 patients; 3%) were identified as the most frequent PSIs and HACs. PSIs were present in 1 out of 41 patients (5%) for the temporal GTR and 2 out of 20 patients for the lobectomy group (p = 1.0). Respective rates for PSIs were 5 of 41 (12%) and 1 of 20 (5%) (p = 0.7). Further, CSCs did not yield significant differences between these two resection modalities (p = 1.0).

**Conclusion:**

With regard to ATL and GTR as differing onco-surgical approaches these data suggest ATL in terms of an aggressive supra-total resection strategy to preserve perioperative standard safety metric profiles.

## Introduction

In regard of the fatal micro-invasive growth pattern of glioblastoma with histological evidence of malignant cells distant from magnetic resonance imaging (MRI)-detectable abnormalities, the perception of extended resection regimes far beyond the enhancing tumor bulk lesions increasingly is evolving within the field of surgical neuro-oncology [1–4]. Thus, extended supra-marginal glioblastoma resection should provide for superior long-term disease control and several studies have reported a survival benefit in these extended-resected patient cohorts, so far [5–7]. Recently, it has been demonstrated that in temporal glioblastoma disease resection of the entire anterior temporal lobe as a maximum variant of such supra-total conceptual approaches yielded significant prolonged median progression-free survival (mPFS) and median overall survival (mOS) rates therefore suggesting anterior temporal lobectomy (ATL) to constitute the surgical modality of choice for temporal-located glioblastoma [8]. However, given a potential increased impairment of eloquent areas and critical vasculature at risk, supra-marginal resection regimes might be accompanied by elevated levels of postoperative unfavorable events which in turn significantly worsen patients’ postoperative quality of life [9]. Against this backdrop the present study was aimed at comparatively analyzing temporal GTR and ATL as differing onco-surgical resection modalities with regard to the onset of early postoperative unfavorable events in the course of temporal glioblastoma surgery.

## Methods

### Patients and study design

All patients aged ≥ 18 years that underwent surgical resection of newly diagnosed temporal glioblastoma at the authors’ department were entered in a computerized database (SPSS, version 25, IBMCorp., Armonk, NY). Surgical resection strategies were divided into the group of temporal gross total resection of the contrast-enhancing tumor manifestation as well as the group of anterior temporal lobectomy. In case of temporal lobectomy as a supra-total onco-surgical resection strategy, the posterior margin of the resection was approximately 5–6 cm from the temporal tip on the non-dominant hemisphere, and 4–5 cm on the dominant side as described as a highly-standardized protocol for temporal lobe epilepsy surgery [10]. With regard to the retrospective study design, determination of temporal GTR or ATL as differing neurosurgical resection procedures was primarly based upon the prevalence of the particular neurosurgeon. Thus, ATL was performed only by neurosurgeons that were familiar to epilepsy surgical skills. Every neurosurgeon involved in the surgical treatment of the patients included in the present series fulfilled the requirements for Neuro-Oncology Centers certificated by the German Cancer Society. Patients with temporal glioblastoma were only included if the temporal tumor manifestation was precisely within the range of 4–5 cm on the dominant hemisphere and 5–6 cm on the nondominant hemisphere from the temporal tip including those with additional temporomesial infiltration (hippocampus, amygdala, parahipocampal gyrus and/or entorhinal cortex) objectified by means of preoperative gadolinium enhanced MR-imaging with identical sequence programs on a 3.0-T scanner (Achieva TX, Philips Healthcase, Best, Netherlands). Extent of resection was determined based on gadolinium-enhanced MR-imaging within 72 h after surgery. Gross total resection was defined as a complete resection without any residual nodular enhancement. Only patients with GTR or anterior temporal lobectomy were included for further analysis. Histopathological grading was performed according to the 2016 WHO criteria [11]. MGMT promotor methylation status was analyzed using pyrosequencing and combined bisulfite restriction analysis [12]. This study was in compliance with the Helsinki Declaration and approved by the local institutional ethics committee. In regard to the retrospective study design, ethical approval was granted without the need for obtaining patient consent.

Pertinent clinical information including coexistence of arterial hypertension, coronary artery disease, atrial fibrillation, diabetes mellitus, antiplatelet and anticoagulant medication intake prior to surgery as well as history of pulmonary embolism (PE)/deep vein thrombosis (DVT) were assessed and further analyzed. Karnofsky performance scale (KPS) was used to grade patients’ functional status at admission. Further stratification was made by means of the American Society of Anesthesiologists (ASA) classification into a group with preoperative ASA 1 or 2 as well as a group with ASA 3 or 4.

### Screening for postoperative complications

**Fig. 1:**
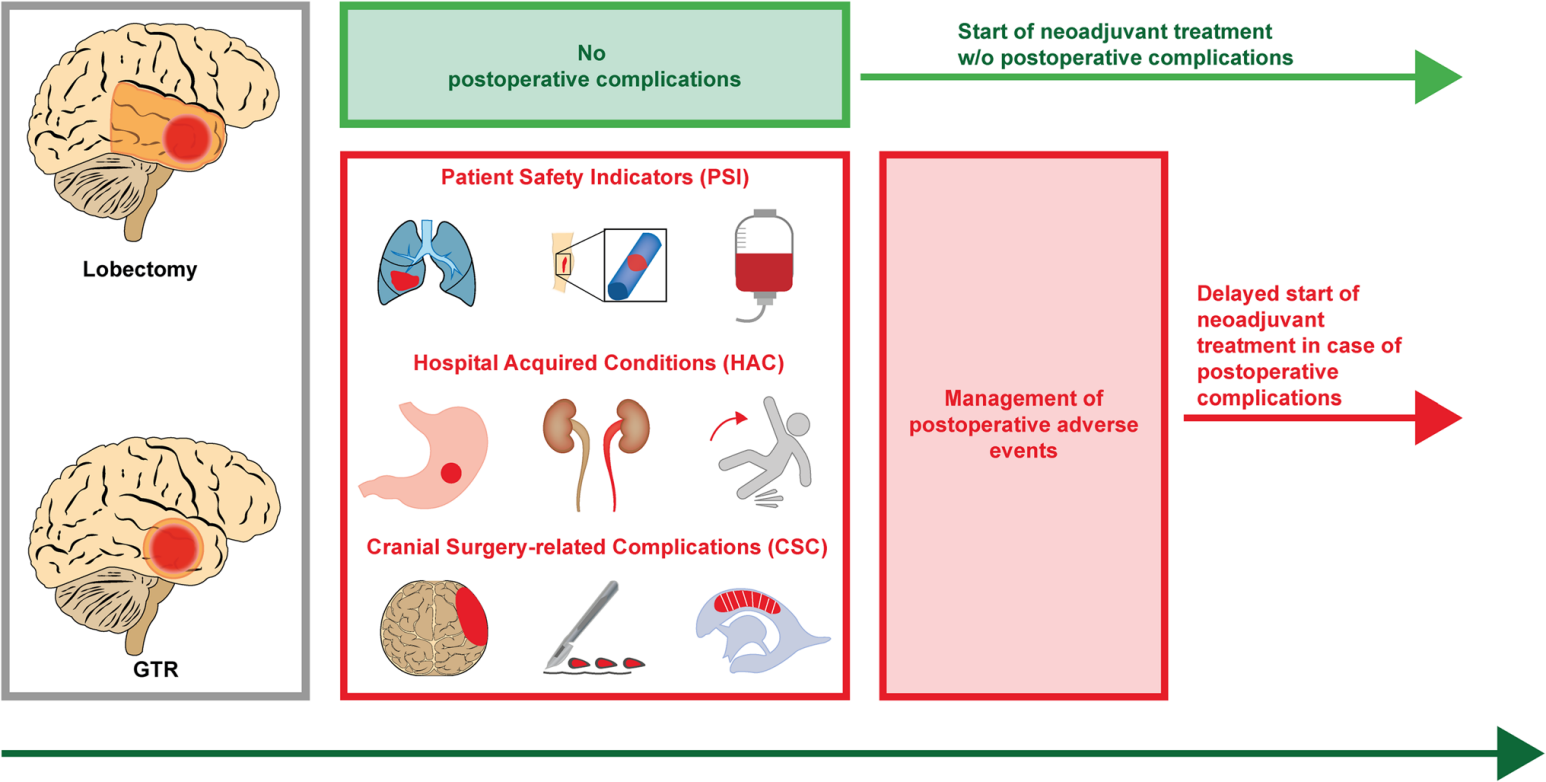
Graphical synopsis of safety metric profiling in temporal glioblastoma surgery

In order to reach for highly standardized quality metric rating and profiling, analysis was done by means of a public-available list of events termed patient safety indicators (PSIs) and hospital-acquired conditions (HACs) introduced by the Agency of Healthcare Research and Quality and the Center for Medicare and Medicaid Services (Fig. 1) [13, 14]. Thereby, PSIs entailed pressure ulcer, iatrogenic pneumothorax, central venous catheter-related blood stream infection, transfusion reaction, retained surgical item, peri- and postoperative hemorrhage, acute postoperative respiratory failure, pulmonary embolism, deep venous thrombosis (DVT), postoperative sepsis, wound dehiscence, accidental puncture or laceration, postoperative hip fracture as well as postoperative physiologic and metabolic derangement. Within the group of HACs, screening was performed for foreign object retained after surgery, air embolism, blood incompatibility, pressure ulcer states II and IV, catheter-associated urinary tract infection, fracture, dislocation, intracranial injury, crushing injury, vascular catheter-associated infection and manifestation of poor glycemic control (diabetic ketoacidosis, nonketonic hyperosmolar coma, hyperglycemic coma). Postoperative periods were additionally screened for iatrogenic ischaemic infarction, cerebro spinal fluid (CSF) leakage, postoperative meningitis and ventriculitis as well as postoperative new or worsened neurological deficits including postoperative new speech and language deficits and subsequently classified as specific cranial surgery-related complications (CSCs). Occurrence of early postoperative complications served as primary readout and was defined as any postoperative unfavorable event that appeared within 30 days following initial temporal glioblastoma resection.

### Statistics

Data analyses were performed using the computer software package SPSS (version 25, IBM Corp., Armonk, NY). Categorical variables were analyzed in contingency tables using Fisher’s exact test. For discrete and continuous variables mean and standard deviation (SD) was determined unless stated otherwise. Results with p < 0.05 were considered statistically significant.

## Results

### Baseline characteristics

Between 2012 and 2018, 61 patients were surgically treated for temporal-located glioblastoma at the authors’ institution. Preoperative MR-imaging revealed tumor manifestation located precisely within the temporal lobe in 39 patients (64%), whereas 22 patients (36%) exhibited additional temporomesial malignant infiltration. Anterior temporal lobectomy and temporal GTR were performed in 20 (33%) and 41 patients (67%), respectively. Overall, early postoperative unfavorable events following resection of temporal glioblastoma were present in 10 out of 61 patients (16%). For further details see Table 1. Patients with GTR exhibited a mOS rate of 12 months (95% CI 5–14) compared to 23 months (95% CI 16–33) for patients with ATL (p = 0.0005).

**Table 1:** Baseline characteristics

Sex	n
Male	39 (64)
Female	22 (36)
Mean age (± SD) (in yrs)	63 ± 13
Tumor location	
Temporomesial infiltration (−)	39 (64)
Temporomesial infiltration (+)	22 (36)
Preoperative KPS	
≥ 70	47 (77)
< 70	14 (23)
Surgical modality	
Temporal lobectomy	20 (33)
Temporal GTR	41 (67)
MGMT promotor methylation status	
Methylated	22 (43)
Unmethylated	29 (57)
IDH mutation status	
Wild type	37 (97)
Mutant	1 (3)
Postoperative complications	10 (16)

Values represent number of patients unless otherwise indicated (%)

*ds* days, *GTR* gross total resection, *KPS* Karnofsky performance scale, *SD* standard deviation, *yrs* years

### Early postoperative complications

Analysis of early postoperative complications revealed subclassification into 6 PSIs, 2 HACs as well as 2 specific CSCs (Table 2). Postoperative hemorrhage and catheter-associated urinary tract infection were the most frequent PSIs and HACs accounting for 5% and 3% of patients within the overall cohort of temporal glioblastoma patients, respectively. Among the patients with postoperative hemorrhage, revision surgery was required for one subject. Postoperative sepsis, wound dehiscence and pulmonary embolism were identified as further PSIs in 1 out of 61 patients (2%) each. The subgroup of specific CSCs was made up of postoperative CSF leakage and meningitis accounting for 1 patient (2%), respectively. No profound new language or visual deficit was seen within the routine follow up examinations.

**Table 2:** Overview of postoperative complications

No. of patients	61
No. of complications	10 (16)
PSIs	6 (10)
Postoperative hemorrhage	3 (5)
Postoperative sepsis	1 (2)
Wound dehiscence	1 (2)
Perioperative pulmonary embolism	1 (2)
HACs	2 (3)
Catheter-associated urinary tract infection	2 (3)
Specific CSCs	2 (3)
CSF leakage	1 (2)
Meningitis	1 (2)

Values represent number of patients (%) unless otherwise indicated

*CSCs* cranial surgery-related complications, *CSF* cerebro spinal fluid, *HACs* hospital-acquired conditions, *No.* number of patients, *PSIs* patient safety indicators

### Safety metric profiling of ATL and temporal GTR as differing onco-surgical resection modalities

In order to sufficiently compare safety metric profiles of anterior temporal lobectomy and temporal GTR by means of postoperative complication rates, both strategies of temporal glioblastoma resection were first analyzed for potential patient- and tumor-related covariates. Comparative analysis of preoperative KPS, KPS at discharge and ASA-scoring as well as comorbidities such as arterial hypertension, coronary artery disease and diabetes mellitus among others revealed homogeneous distribution of risk factor profiles in both these groups of differing resection strategies (Table 3). Similarly, presence of temporomesial malignant infiltration, mean operation time, mean hospital stay and postoperative new seizure onset within 3 months after surgery were evenly distributed. Volumetric analysis of preoperative tumor and postoperative resection cavity volumes could objectify intended supratotal extent of resection for anterior temporal lobectomies: while mean resection cavity volumes revealed 29 cm^3^ for temporal GTR versus 58 cm^3^ for temporal lobectomy (p = 0.0001), preoperative tumor volumes did not significantly differ for these two resection modalities (31 cm^3^ vs. 33 cm^3^, p = 0.8).

**Table 3:** Safety metric profiling of ATL and temporal GTR

	Temporal lobectomy 20 (33)	Temporal GTR 41 (67)	p value
Mean age (yrs)	61 ± 12	63 ± 12	0.2
Sex			0.6
Female	6 (30)	16 (39)	
Male	14 (70)	25 (61)	
KPS preoperative			1.0
≥ 70	16 (80)	31 (76)	
KPS at discharge			
≥ 70	15 (80)	29	1.0
Tumor location			0.3
Temporomesial infiltration (−)	15 (75)	24 (59)	
Temporomesial infiltration (+)	5 (25)	17 (41)	
Volumetric analysis			
Mean tumor volume	33 ± 21	31 ± 25	0.8
Mean resection cavity volume	58 ± 15	29 ± 19	0.0001
Mean operation time (min)	270 ± 97	268 ± 67	0.9
Mean hospital stay (ds)	14 ± 7	15 ± 8	0.6
Postoperative seizure onset^a^	0 (0)	2 (5)	1.0
ASA score			1.0
1 or 2	15 (75)	31 (76)	
3 or 4	5 (25)	10 (24)	
Anticoagulant medication prior to surgery	4 (20)	4 (10)	0.4
Comorbidities			
Arterial hypertension	5 (25)	16 (39)	0.4
Coronary artery disease	2 (10)	1 (2)	0.2
Atrial fibrillation	1 (5)	1 (2)	1.0
Diabetes mellitus	1 (5)	4 (10)	1.0
History of PE/DVT	1 (5)	1 (2)	1.0
Postoperative complications			
PSIs	1 (5)	5 (12)	0.7
HACs	1 (5)	1 (2)	1.0
Specific CSCs	1 (5)	1 (2)	1.0

Values are presented as the number of patients (%) unless stated otherwise

*ASA* American Society of Anesthesiology, *ATL* anterior temporal lobectomy, *CSCs* cranial surgery-related complications, *ds* days*, DVT* deep vein thrombosis*, GTR* gross total resection, *HACs* hospital-acquired conditions, *KPS* Karnofsky Performance Scale*, min,* minutes, *No.* number, *PE* pulmonary embolism, *PSIs* patient safety indicators, *yrs* years

^a^Within 3 months after surgery

Having demonstrated equal distribution of potential interfering cofounders, ATL and temporal GTR subsequently were compared for the rate of postoperative early unfavorable events. PSIs were present in 5 out of 41 patients (12%) for the temporal GTR and 1 out of 20 patients (5%) for the lobectomy group (p = 0.7) (Table 3). Respective rates for HACs were 1 out of 41 (2%) and 1 out of 20 (5%) (p = 1.0). Analysis of specific cranial CSCs yielded 1 out of 41 (2%) patients with postoperative CSF-leakage in the GTR group as well as 1 out of 20 patients (5%) with postoperative meningitis in the lobectomy group (p = 1.0). In summary, PSIs, HACs and specific CSCs did not significantly differ between these two groups of differing onco-surgical approaches.

## Discussion

Given the growing evidence of a greater extent of malignant primary brain tumor resection to entail significantly prolonged time to tumor progression and superior overall survival rates, the concept of supra-total resection strategies far beyond tumoral enhancing MRI abnormalities is increasingly emerging in the field of neurosurgical oncology [6, 15–17]. In a recent study, we could demonstrate that—in temporal glioblastoma disease—ATL as a paradigm for such supra-marginal extended surgery regimes was accompanied by a significant survival benefit compared to conventional gadolinium- and 5-ALA-guided temporal GTRs [8]. However, the aim of aggressive extended surgical approaches has to surrender the risk of potential elevated levels of postoperative complications [9]. Patients who suffer from postoperative unfavorable events following malignant brain tumor resection have been shown to exhibit profound worsened long-term quality of life as well as to be at a more than 4-times higher risk for in-hospital mortality [9, 18]. Furthermore, postoperative complications have been reported to be accompanied by a delay of adjuvant chemotherapy therefore additionally reducing oncological outcomes (Fig. 1) [19, 20]. In the present study, we therefore analyzed ATL and temporal GTR as differing onco-surgical resection modalities for temporal-located glioblastoma in view of the incidence of postoperative early complications. In order to achieve a comprehensive assessment of overall postoperative complication rates in terms of highly standardized quality metric profiling, screening for postoperative events was performed by means of PSIs and HACs as public-available quality rating classification tools [13, 14].

The overall morbidity rate was found to reach about 16%. Thereby, the quantitative complication level in our series was in accordance to existent literature where respective values on overall complications in glioblastoma surgery are given between 11 and 32% [16, 21–23]. It is important to mention that a PSI- and HAC-based assessment of postoperative unfavorable events will also cover transient events like catheter-associated urinary tract infections and therefore will quantitatively surpass complication levels of several previous studies that focused exclusively on postoperative events which required further surgical treatment [21, 24, 25]. Further, postoperative secondary hemorrhage and urinary tract infection were identified as the most common unfavorable events within the presented patient cohort of selected temporal glioblastoma disease and observed levels of incidence met previously reported data [26–28].

Presented analysis of postoperative complications dependent on the surgical modality within temporal glioblastoma surgery did not reveal any indications to increased risk profiles in case of supra-total temporal glioblastoma resection. As against several concerns that have been raised on potential elevated risks in the context of far extended tumor resections, similar findings of unimpaired or even decreased levels of iatrogenic postoperative complications following supra-marginal glioblastoma resection have been reported for cohorts of topographically unselected glioblastoma locations [27]. Such preservation of quality metric profiles in the course of supra total glioblastoma resection might—among others—be reasoned in a reduced risk for the development of postoperative reactive peritumoral edema as well as a decreased risk of postoperative intratumoral bleeding from partially-resected vulnerable tumor remnants [27, 29]. The current study is the first to provide data on postoperative complications of supra-total tumor resection in the setting of specifically temporal-located glioblastoma disease. In contrast to the inconsistent perception of the extent of supra-total tumor removal within the existing literature varying from resection beyond T1-enhanced tumor areas, but within the boundaries of FLAIR abnormalities, to resection beyond any visible MRI abnormality, ATL as a maximum variant of a supra total resection policy with excision of the entire anterior temporal lobe constitutes a highly-standardized and clearly-defined procedure [8, 30, 31]. Therefore, subsequent rather multicenter studies will be capable not only to cope with the low frequency of temporal glioblastoma patients, but also to sufficiently evaluate the potential of ATL by means of intercenter comparison to supersede a mere complete tumor bulk removal and thus constitute the surgical modality of choice for temporal-located glioblastoma.

## Limitations

The present study has several limitations. Acquisition of data was retrospective and therefore, determination of the surgical resection modality was based on the preference of the particular neurosurgeon and not randomized. Owed to the retrospective nature, a comprehensive assessment of visual field deficits by ophthalmologic examinations as well as specific language assessment tools were beyond the scope of the present manuscript. Further, though not statistically significant, given the trend of a higher rate of preoperative tumoral temporomesial infiltration within the ATL group, determination of the surgical resection modality might potentially not only be associated with neurosurgeon-specific preferences, but also with characteristics of tumor location. Given the highly-selective inclusion criteria of temporal-located glioblastoma patients, the study population was quite small and therefore might entail unconsidered further selection bias. Future multicenter studies might clarify such potential cofounding restraints. Additionally, the present data represent a single-center experience, only.

## Conclusions

With regard to ATL and GTR that did not exhibit significant differences in the rates of peri- and postoperative unfavorable events in the course of temporal glioblastoma resection, our data suggest ATL in terms of a supra-total conceptual resection modality not to fail perioperative standard safety metric profiles. Given the previously reported survival benefit compared to a mere complete tumor bulk removal, ATL might constitute the surgical modality of choice for temporal-located glioblastoma.

## Data Availability

Restrictions apply to the availability of these data due to privacy restrictions.
